# Nanoantenna Structure with Mid-Infrared Plasmonic Niobium-Doped Titanium Oxide

**DOI:** 10.3390/mi11010023

**Published:** 2019-12-24

**Authors:** Hai Dang Ngo, Kai Chen, Ørjan S. Handegård, Anh Tung Doan, Thien Duc Ngo, Thang Duy Dao, Naoki Ikeda, Akihiko Ohi, Toshihide Nabatame, Tadaaki Nagao

**Affiliations:** 1 International Center for Materials Nanoarchitectonics, National Institute for Materials Science, Tsukuba 305-0044, Japan; NGO.HaiDang@nims.go.jp (H.D.N.); kaichen@jnu.edu.cn (K.C.); HANDEGARD.Orjansele@nims.go.jp (Ø.S.H.); DOAN.TungAnh@nims.go.jp (A.T.D.); DUCTHIEN.Ngo@nims.go.jp (T.D.N.); katsiusa@gmail.com (T.D.D.); IKEDA.Naoki@nims.go.jp (N.I.); OHI.Akihiko@nims.go.jp (A.O.); NABATAME.Toshihide@nims.go.jp (T.N.); 2 Department of Condensed Matter Physics, Graduate school of Science, Hokkaido University, Kita-10 Nishi-8 Kita-ku, Sapporo 060-0810, Japan; 3 Institute of Photonics Technology, Jinan University, Guangzhou 510632, China; 4 Nanotechnology Innovation Station, National Institute for Materials Science, Tsukuba 305-0044, Japan

**Keywords:** nanoantenna, niobium-doped titanium oxide, mid-infrared plasmonics

## Abstract

Among conductive oxide materials, niobium doped titanium dioxide has recently emerged as a stimulating and promising contestant for numerous applications. With carrier concentration tunability, high thermal stability, mechanical and environmental robustness, this is a material-of-choice for infrared plasmonics, which can substitute indium tin oxide (ITO). In this report, to illustrate great advantages of this material, we describe successful fabrication and characterization of niobium doped titanium oxide nanoantenna arrays aiming at surface-enhanced infrared absorption spectroscopy. The niobium doped titanium oxide film was deposited with co-sputtering method. Then the nanopatterned arrays were prepared by electron beam lithography combined with plasma etching and oxygen plasma ashing processes. The relative transmittance of the nanostrip and nanodisk antenna arrays was evaluated with Fourier transform infrared spectroscopy. Polarization dependence of surface plasmon resonances on incident light was examined confirming good agreements with calculations. Simulated spectra also present red-shift as length, width or diameter of the nanostructures increase, as predicted by classical antenna theory.

## 1. Introduction

In recent times, infrared nano-plasmonics shifts its interest from common noble metals or compounds like Au, Pt, Ag or TiN [1] to transparent conductive oxides as materials of choice [2]—namely, indium tin oxide [3,4,5,6,7,8,9], fluorine doped tin oxide [10,11], aluminum (or gallium) doped zinc oxide [12,13,14,15,16,17]. Extensive research on those materials aims at both basic properties in infrared region as well as different applications such as wavelength-selective perfect absorbers and emitters [6,18,19] interacting plasmonic nano-particles [20,21,22,23], optical meta-surfaces [19], and active tunable plasmonic devices [7,19,24]. Compared to those oxide materials, niobium-doped titanium oxide (TiO_2_:Nb) exhibits notable electrical and optical properties. The so-called name “transparent metal” comes from high transparency in visible light and noticeably low electrical resistivity. It also has great thermal durability, high surface smoothness, mechanical robustness, humid environment endurance and other advantages, just to name a few [25,26,27,28,29,30,31,32].

In this report, we demonstrate the use of TiO_2_:Nb in nanostrip and nanodisk antenna arrays for infrared plasmonic devices such as surface-enhanced infrared absorption spectroscopy (SEIRA). The nanopatterned arrays were fabricated using electron beam lithography combined with plasma etching and oxygen plasma ashing processes. The fabricated nanostrip and nanodisk antenna structures of TiO_2_:Nb were sequentially subjected to linearly polarized infrared light in relative transmittance measurement with Fourier transform infrared spectroscopy. Resulted plasmon resonances exposed polarization dependence and shifted to longer wavelengths as the length, width of nanostrips, or diameter of nanodisks expanded. Experiment and simulation results are in good concordance.

## 2. Materials and Methods

**Figure 1:**
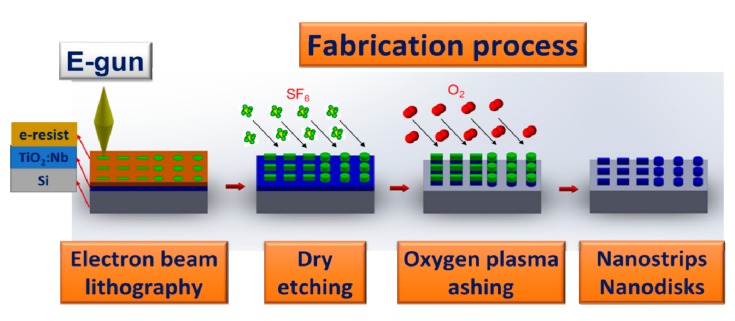
Fabrication process of Nb-doped TiO_2_ nanoantenna (from left to right) consists of electron beam lithography, development, dry etching with SF_6_ gas, and oxygen plasma ashing. The electron resist layer (**brown**) was coated on TiO_2_:Nb thin film (**blue**), which was deposited on silicon substrate (**grey**). The remained electron resist layer (**green**) after lithography process helped to define the nanostrip and nanodisk of antenna structures.

Fabrication process of niobium-doped titanium dioxide nanoantenna is demonstrated generally in Figure 1.

At first, the Nb-doped TiO_2_ film was co-sputtered at room temperature with TiO_2_ ceramic and Nb metallic targets. Both silicon (001) and borosilicate glass were used simultaneously as sputtering substrates in every deposition. Radio frequency (RF) and direct current (DC) sputtering methods were used for TiO_2_ and Nb targets, respectively. The sputtering chamber (!-Miller, Shibaura Mechatronics Corporation, Yokohama, Japan) was initially evacuated down to 4.0 × 10^−5^ Pa as base pressure, then 19 sccm of argon and 1 sccm of oxygen gas flow was introduced into the chamber to create working pressure of about 0.3 Pa. Sputtering power was set at 200 W (RF) and 20 W (DC) for TiO_2_ and Nb targets, respectively. After one hour of deposition, 80-nm-thick film was obtained. Then, the as-deposited films went through vacuum thermal annealing at 600 °C for 1 h. Optical properties of TiO_2_:Nb thin films were characterized with Ellipsometry (SENTECH, SE 850 DUV and SENDIRA, SENTECH Instruments GmbH, Berlin, Germany) from deep ultraviolet to far infrared region.

Consequently, the samples were spin coated with negative resist NBE-22A for electron beam lithography (at 2000 rounds per minute in 60 s) and baked with hot plate (110 °C in 5 min). Electron beam lithography (ELIONIX, ELS-7500EX, ELIONIX INC, Tokyo, Japan) was used to write desired pattern onto the resist layer (Figure 1). After pattern writing process, samples were subjected to post-baking (110 °C in 5 min), developing with NMD-3 in 60 s and rinsing with iso-propyl alcohol (IPA), and finally blow-drying with nitrogen gas gun.

Reactive ion etching (ULVAC, CE-300I, SF_6_ gas, 0.5 Pa, 100 W power) and oxygen plasma ashing (Mory PB-600, 300 W power in 15 min) steps were carried out to remove unnecessary surrounding TiO_2_:Nb nanostrips or nanodisks and wash away the remaining electron resist.

Shape and morphology of strip and disk nanostructures were investigated with scanning electron microscopy (SEM, Hitachi, SU-8400, Hitachi High-Technologies Corporation, Tokyo, Japan) and Atomic force microscope (AFM, Nanoscope 5, Bruker Corporation, Billerica, MA, USA). The silicon tips SI-DF20 (Hitachi High Technologies, Tokyo, Japan) were used at tapping mode. 

**Figure 2:**
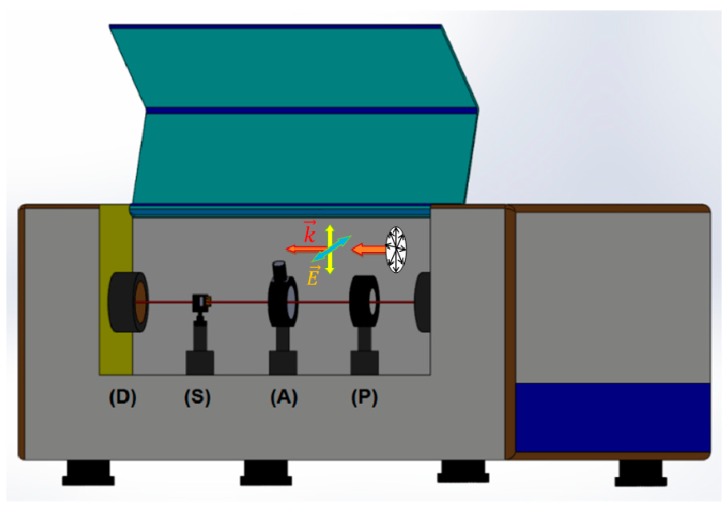
Transmission measurement setup with Fourier transform infrared spectrometer. Unpolarized incident infrared light (right side) passed through polarizer (**P**), aperture (**A**) and then exposed to nanostrip sample (**S**) before entering the detector (**D**). In case of nanodisk sample, polarizer (**P**) was removed and unpolarized infrared light was used.

Fourier transform infrared spectroscopy (FTIR, Thermo Scientific Nicolet iS50, Waltham, MA, USA) was used to assess the resonance features of nanostructures with different linear polarization of incident light, i.e., electric field vector was either parallel to length ($\vec{E_{∥}}$) or width ($\vec{E_{⊥}}$)of nanostrips, as illustrated in Figure 2. Unpolarized incident light was used for nanodisks.

## 3. Results and Discussion

**Figure 3:**
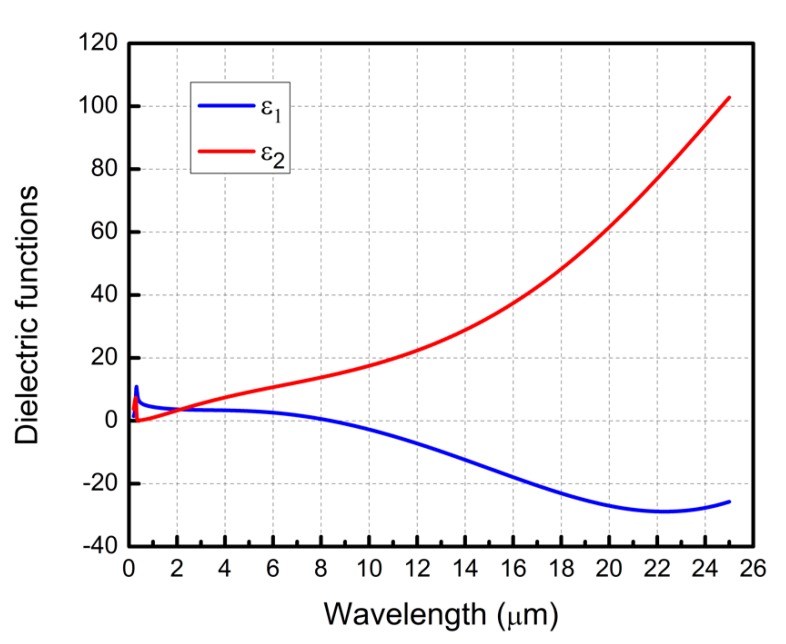
Experimental dielectric functions of TiO_2_:Nb films on silicon substrate. Real (ε_1_) and imaginary (ε_2_) parts in blue and red curves, respectively.

Dielectric functions (ε_1_, ε_2_) of co-sputtered Nb-doped TiO_2_ films on silicon substrate (001) were characterized with spectroscopic ellipsometry at three different reflected angles of 60°, 65°, and 70° from ultraviolet (0.3 μm) to far infrared (25 μm) region (Figure 3).

Real part of the dielectric function (ε_1_) exhibited a cross-over point in the mid-infrared region at approximately 8.5 µm. By increasing the Nb sputtering power, carrier concentration can be tuned, and Fermi level can easily be shifted toward conduction band. This in turn helps to adjust the cross-over point back and forth in the infrared region, whereas for conventional plasmonic metals such continuous tunability is not possible. Furthermore, this material also has great thermal durability, high surface smoothness, mechanical robustness, as well as photocatalytic activity and humid environment endurance. Combining altogether, TiO_2_:Nb film proves itself an excellent candidate for diverse infrared plasmonic applications.

**Figure 4:**
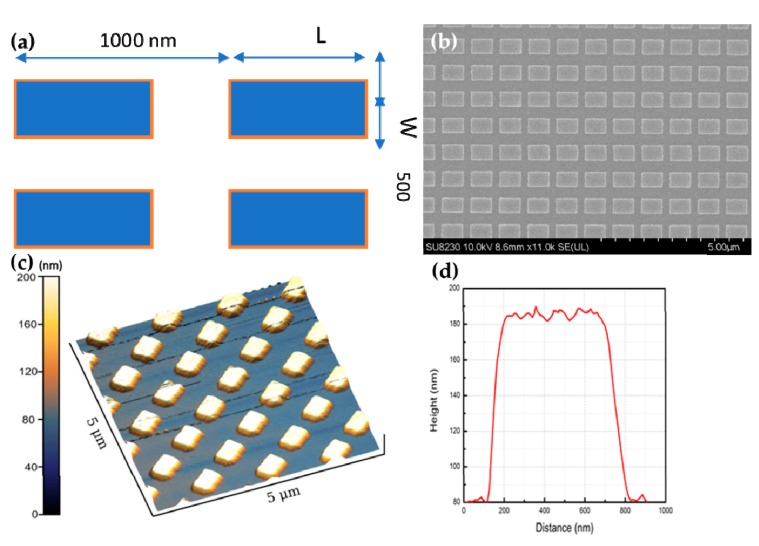
Design patterns of TiO_2_:Nb antenna for simulation and electron beam lithography (**a**). Horizontal periodicity is 1000 nm. Strip width (W) is about 500 nm. SEM image of TiO_2_:Nb nanostrips (**b**). Three-dimensional AFM image (5 μm × 5 μm) of TiO_2_:Nb nanostrips (**c**). Height profile of a single nanostrip (**d**).

To realize the plasmonic property of co-sputtered TiO_2_:Nb films in infrared region, nanostrips of different sizes were simulated with electromagnetic solver to check for the antenna resonance. The simulated parameters were transferred into lithographic patterning and fabrication, and then characterized by the IR spectroscopy. Figure 4a showed general designed nanostrip pattern used for Finite-Difference Time-Domain method (FDTD, Rsoft, Synopsis) simulation as well as electron beam lithography. In simulation, the lengths (L) of the strips were set from 650 to 750 nm while the width values (W) varied from 400 to 600 nm.

SEM and AFM images (Figure 4b,c) show periodic rectangular nanostrips, which are similar to intended design pattern (Figure 4a). The average strip surface roughness of 2.3 nm confirms that the etching and ashing processes did not cause considerable damage.

**Figure 5:**
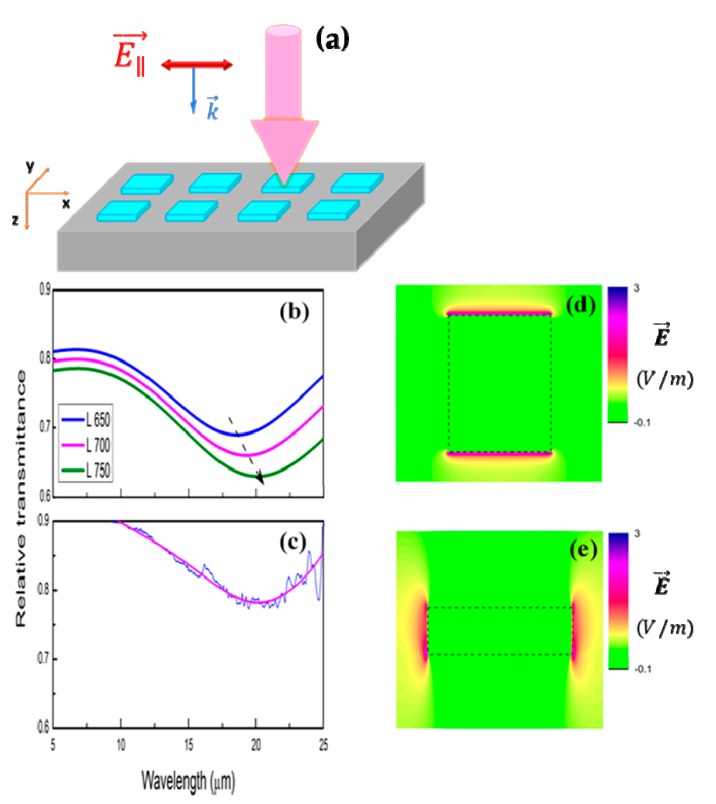
Schematic illustration of the exposure of polarized infrared light with electric field vector (k) parallel to length of nanostrips ($\vec{E_{∥}}$) (**a**). Simulation (**b**) and FTIR relative transmittance (**c**) of nanostrips with ($\vec{E_{∥}}$) polarized incident light. Lengths of nanostrips (L) in simulation were set at 650, 700, and 750 nm, corresponding to blue, pink, and green curves (**b**). Measured and fitted lines were drawn in blue and pink, respectively (L × W ~ 750 nm × 500 nm) (**c**). Electric field distribution along x–y and x–z planes of strips (**d**, **e**).

**Figure 6:**
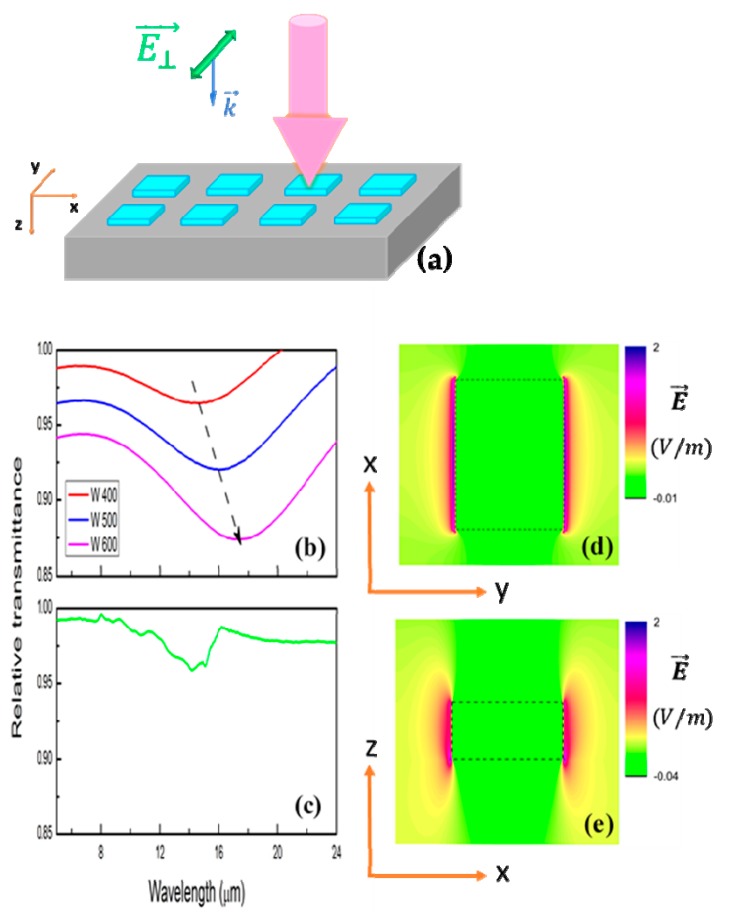
Schematic illustration of polarized infrared light with electric field vector parallel to the longer axis of nanostrips ($\vec{E_{⊥}}$) (**a**). Simulation (**b**) and FTIR relative transmittance (**c**) of nanostrips with ($\vec{E_{⊥}}$) polarized incident light. Widths of nanostrips (W) in simulation were set at 400, 500, and 600 nm, corresponding to red, blue, and pink curves (**b**). Measured spectrum was plotted in green line (L × W ~ 700 nm × 450 nm) (**c**). Electric field distribution along x-y and x-z planes of strips (**d**, **e**).

The smallest interval between two consecutive fabricated strips is approximately 300 nm. As illustrated in Figure 5d and Figure 6d, relative transmittance exhibits strong fundamental antennalike resonances as polarized electric field vector is parallel to nanostrip length or width. Both measured and simulated spectra show good agreement. Calculated spectra demonstrate orderly shift of resonances toward longer wavelengths (black dash arrow) and reveal the improvement in quality factor of nanoantenna (sharper and deeper resonances) as length or width of nanostrips increases, as in the case of metal nanowires [32]. FDTD simulation results along x–y and x–z planes (Figure 5d,e and Figure 6d,e) also show strong electric field confinement and enhancement at the edges of nanostrips. This proves the antennalike dipole resonance of the structures.

**Figure 7:**
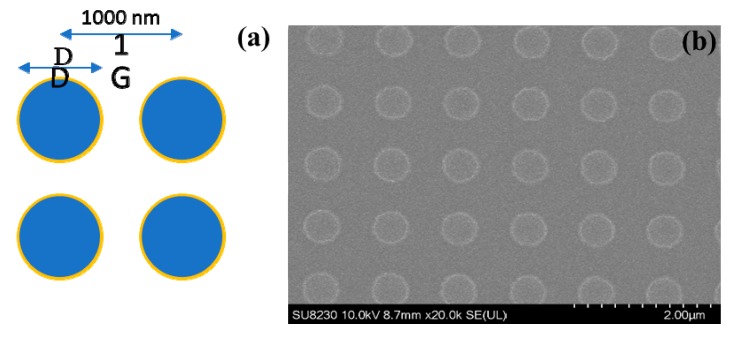
Design patterns of TiO_2_:Nb nanodisk antennas for calculation and fabrication. Periodicity is 1000 nm (**a**). SEM image of TiO_2_:Nb nanodisk structure (**b**).

General designed nanodisk pattern for FDTD calculation and electron beam lithography was shown in Figure 7a. Diameters of nanodisks (D) were set from 600 to 800 nm.

**Figure 8:**
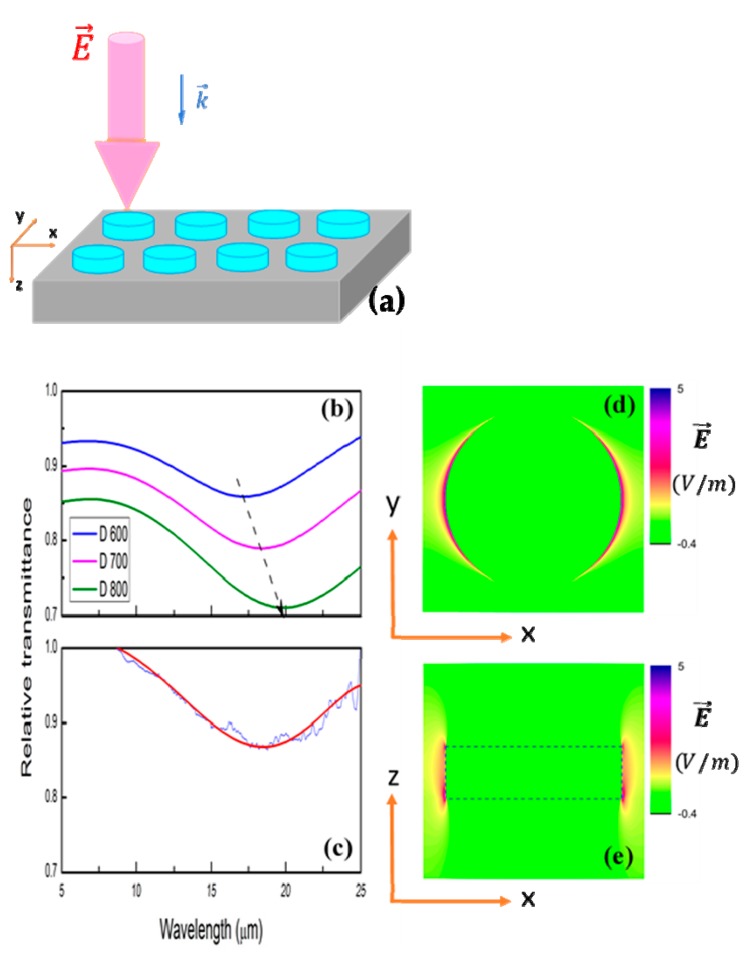
Schematic diagram of unpolarized infrared light on nanodisk antenna structures (**a**). Simulation (**b**) and FTIR relative transmittance (**c**) of nanodisk with unpolarized incident light. Diameters of nanodisks (D) in simulation were set at 600, 700, and 800 nm, corresponding to blue, pink, and green curves. Measured and fitted lines were drawn in blue and red (D ~750 nm) (**c**). Electric field distribution along x–y and x–z planes (**d**, **e**).

In simulated results, systematic increase of resonance frequency with diameter (D) can be easily observed. Sharper resonances can be expected if disk diameter keeps expanding. Experimental relative transmittance spectrum is in consonance with calculated lines with strong fundamental antennalike resonances under any linear polarized infrared light parallel to the disk surface (Figure 8d). Quality factor of these nanodisk antennae can be further improved when the diameter D increases since the surface plasmon resonance assumes more photonic nature and the inherent loss of the material becomes less. While resonant frequencies show good matching between simulation and experiment curves in x axis; there is some difference (~10% to 15%) in relative transmittance (*y*-axis). This can be explained by two factors: fabrication tolerance and etchant effect. Perfect cuboids were used in simulation, but the obtained ones were not perfectly flat at edges, as shown in Figure 4c,d. Furthermore, electron carrier concentration was decreased from 1.12 × 10^21^ cm^−3^ for pristine film to 6.4 × 10^20^ cm^−3^ for remained one. SF_6_ gas may introduce some electron-trapping fluorine ions on surface of nanostructure after etching.

## 4. Conclusions

We succeeded in designing and fabricating antenna arrays of nanostrips and nanodisks based on Nb doped TiO_2_. Relative infrared transmittance in calculated and experimental results are in good accordance. Spectral features exhibit red-shift in their peak position as the sizes of strips and disks increase. In the same tendency, quality factor of nanostrips and nanodisks can also be improved if structure dimension can be further extended.
